# MorphoCatcher: a multiple-alignment based web tool for target selection and designing taxon-specific primers in the loop-mediated isothermal amplification method

**DOI:** 10.7717/peerj.6801

**Published:** 2019-04-26

**Authors:** Fedor V. Shirshikov, Yuri A. Pekov, Konstantin A. Miroshnikov

**Affiliations:** 1 Shemyakin & Ovchinnikov Institute of Bioorganic Chemistry RAS, Moscow, Russia; 2 Lomonosov Moscow State University, Moscow, Russia

**Keywords:** LAMP, PrimerExplorer, Target selection, Gene polymorphism, Primer ends

## Abstract

**Background:**

Advantages of loop-mediated isothermal amplification in molecular diagnostics allow to consider the method as a promising technology of nucleic acid detection in agriculture and medicine. A bioinformatics tool that provides rapid screening and selection of target nucleotide sequences with subsequent taxon-specific primer design toward polymorphic orthologous genes, not only unique or conserved common regions of genome, would contribute to the development of more specific and sensitive diagnostic assays. However, considering features of the original software for primer selection, also known as the PrimerExplorer (Eiken Chemical Co. LTD, Tokyo, Japan), the taxon-specific primer design using multiple sequence alignments of orthologs or even viral genomes with conservative architecture is still complicated.

**Findings:**

Here, MorphoCatcher is introduced as a fast and simple web plugin for PrimerExplorer with a clear interface. It enables an execution of multiple-alignment based search of taxon-specific mutations, visual screening and selection of target sequences, and easy-to-start specific primer design using the PrimerExplorer software. The combination of MorphoCatcher and PrimerExplorer allows to perform processing of the multiple alignments of orthologs for informative sliding-window plot analysis, which is used to identify the sequence regions with a high density of taxon-specific mutations and cover them by the primer ends for better specificity of amplification.

**Conclusions:**

We hope that this new bioinformatics tool developed for target selection and taxon-specific primer design, called the MorphoCatcher, will gain more popularity of the loop-mediated isothermal amplification method for molecular diagnostics community. MorphoCatcher is a simple web plugin tool for the PrimerExplorer software which is freely available only for non-commercial and academic users at http://morphocatcher.ru.

## Introduction

Loop-mediated isothermal amplification (LAMP) is a method of nucleic acid amplification (Notomi et al., 2000), which was developed to eliminate disadvantages of polymerase chain reaction (PCR) for detection of single nucleotide polymorphisms (SNPs) (Notomi & Hase, 2005).

The LAMP reaction is catalyzed by a *Bst* (*Bacillus stearothermophilus*) DNA polymerase with high tolerance to various sample inhibitors, therefore, the amplification often does not require thorough preparation and purification of target nucleic acids (DNA or RNA). Due to a self-primed principle of amplification and strand displacement activity of *Bst* polymerase, the LAMP reaction can be performed under isothermal conditions (approximately 65 °C) using thermostat instead of expensive thermal cyclers. In contrast to PCR, the LAMP method is very rapid (typically 20–30 min for the reaction), allows to use 4–6 primers (it is equally up to 160 bp of total target coverage by primers), and compatible with different naked-eye detection assays (Goto et al., 2009; Tanner, Zhang & Evans Jr, 2015).

Recently, different LAMP assay formats were developed using kits with lyophilized reagents that can be stored at room temperature (Howson et al., 2017), lab-on-chip approaches based on digital microfluidic technology (Ma et al., 2018), smartphone camera detection systems (Kaarj, Akarapipad & Yoon, 2018), and original technique for the multiplexed detection of target nucleic acids (Ball et al., 2016). Indeed, the complex mechanism of LAMP reaction was very encouraging for many researchers worldwide. Now, LAMP is used for the development of rapid and sensitive assays with simplified sample processing both for laboratory and on-site diagnostics in agriculture and medicine (Notomi et al., 2015; Tomlinson, 2016).

An attractive feature of the LAMP method is that the technology satisfies all required criteria of an ideal diagnostic method proposed by the World Health Organization (Wong et al., 2018). Due to its high speed, specificity, and sensitivity, the method allows to develop a unique assay for intraoperative detection of lymph node metastasis (Tsujimoto et al., 2007). It is obviously that the development of similar PCR-based assay is very complicated because of a number of the PCR method disadvantages. Thus, the LAMP is a promising technique for express and mobile point-of-care testing in different fields of medicine.

Target gene selection is a cornerstone that defines successful primer design and specificity of diagnostic assay. It is known that isothermal conditions of the LAMP reaction impose limits on the genes selected, because of GC-content value of the sequences will affect annealing temperature, primer size, amplicon length, and various thermodynamic parameters of candidate primer sets (e.g., primer dimerization, hairpin formation, and stability of primer ends). An issue of thermodynamic stability in LAMP experiments is usually compounded by the presence of 4–6 primers in the same reaction. Now, many research groups solve the issue by changing the primer set. It is obviously that very small number of genes that will fully meet all the criteria required for optimal amplification. Therefore, a simple solution should be developed for rational target selection and using of the target potential basing on its polymorphic sites, not only conserved common regions. Such approach allows to extract from the target sequence the greatest possible different primer sets with a wide variety of physical-chemical properties.

The PrimerExplorer (Eiken Chemical Co. LTD, Tokyo, Japan; http://primerexplorer.jp/e) is the most widely used software for LAMP primer design (Tomita et al., 2008), that is rapid, easy-to-use, and a free online tool. There are two general primer design modes, namely designing of specific and common primer sets. Currently, the specific designing enables to submit only a single target sequence and select potential annealing sites manually. Unfortunately, manual processing is a time-consuming process and often leads to errors when it deals with large-scale datasets. Moreover, it is not always clear whether the site selected is compatible with target DNA melting and primer annealing temperatures under isothermal amplification conditions. The PrimerExplorer enables to submit MSAs for primer design, but the option is adapted mostly for common primer design. Now, it is possible to establish only conservative regions that could be used as the universal footprints for all closely related species that were involved in the alignment. However, necessary to take into account the conserved species- or group-specific nucleotide variations that look in alignment as empty gaps. Such conserved nucleotide positions, or taxon-specific SNPs, also can be used as the target for primer ends that is a well-known approach for increasing of the amplification specificity.

A number of alternative software for LAMP primer design was suggested, including LAVA (Torres et al., 2011), LAMP Designer (PREMIER Biosoft Int., Palo Alto, CA, USA; http://www.premierbiosoft.com/isothermal/lamp.html), STITCHER (O’Halloran, 2015), FastPCR (Kalendar et al., 2017; PrimerDigital LTD, Helsinki, Finland; http://primerdigital.com/tools/pcr.html), and genome-based LAMP primer design tool like GLAPD (Shanghai Jiao Tong University, Shanghai, China; http://cgm.sjtu.edu.cn/GLAPD). While some of these applications allow to obtain similar primer sets with those from PrimerExplorer, they do not solve the problem with using of nucleotide variations from MSAs for specific primer design. Moreover, LAMP Designer and FastPCR are commercial software, while the LAVA software is no longer available for the LAMP users.

An effective strategy to design specific primers is to use large-scale MSAs of sequences (e.g., highly conserved genes of general biochemical pathways or virulence factors) from multiple strains of the same taxon with subsequent searching of conservative common regions as the potential annealing sites for primers. Then the regions should be verified as taxon-specific sequences using the NCBI BLAST tool (Altschul et al., 1990). This strategy is complicated by the presence of a large number of sequences being orthologous and non-unique for the taxon of interest. Moreover, when we have a set of orthologous targets, the following important questions appear: (i) how to avoid the influence of strain-specific variations on non-specific amplification and (ii) how to use the species-specific polymorphisms for increasing the reaction specificity. It is obvious that the questions could be resolved by using detailed analysis of non-conserved regions in multi-species MSAs. Unfortunately, large multi-species and multi-strain MSAs are not supported as the input in the PrimerExplorer and other LAMP-specialized software. Therefore, a new algorithm is needed to facilitate the MSA analysis, screening of suitable LAMP target among orthologous nucleotide sequences, and successful specific primer design using the PrimerExplorer service.

Another widely used strategy of target selection requires the identification of unique genome region. However, such targets are usually strain-specific and non-conservative for the entire taxon of interest. For instance, some unique virulence factors are usually shared by different bacterial species due to the horizontal gene transfer. Therefore, the unique targets can be useful only for certain diagnostic tasks.

To our knowledge, there is no bioinformatics tool that provides the design of specific LAMP primers using multi-species MSAs, where each species is represented by multiple strains. However, such an application could be beneficial for the LAMP diagnostics community. Our main goal was to design a simple PrimerExplorer web plugin for the users with little or no experience in programming.

Here, we present the MorphoCatcher tool, which allows to select target sequence using a large multi-species alignment, identify all taxon-specific mutations, and use such specific SNPs as a main target for specific primer design option of the PrimerExplorer. The suggested algorithm takes into account the target gene polymorphism and allows to visualize the density of taxon-specific mutations using a sliding-window plot. The MorphoCatcher tool allows to perform coverage of the taxon-specific mutations by primer ends without manual selection of potential annealing sites. The tool is available not only as a web service (http://morphocatcher.ru), but also as a command line Python script.

## Materials and Methods

### Implementation

MorphoCatcher is implemented in Python 3.0 programming language and has a web interface. The algorithm is based on the counting of all taxon-specific mutations, excluding strains-specific polymorphisms, that corresponds to each taxon (or group) in multi-species and multi-strain MSA. Then, a sliding-window function is used to visualize the polymorphism density of potential target for each taxon of the MSA. Thus, MorphoCatcher accelerates the target selection process and allows to select the target region with a high density of taxon-specific mutations.

### Sliding-window approach

To calculate the density of taxon-specific mutations, the following formula of so-called average mutation index was used:

\begin{eqnarray*}& & {\overline{S}}_{f}= \frac{1}{w} \sum _{i=0}^{w-1}{M}_{f-i}= \frac{{M}_{f}+{M}_{f-1}+\cdots +{M}_{f-w+2}+{M}_{f-w+1}}{w} , \end{eqnarray*}

where ${\overline{S}}_{f}$ is the simple moving average (SMA) value for a fragment (*f*) of MSA with a length of 20 nucleotides, which corresponds to the primer annealing site length; *w*—the sliding window size; and *M*_*f*−*i*_—the number of mutations in the fragment (*f*) (*M*_*f*−*i*_ ∈ *N*_0_).

The obtained value of SMA refers to the central fragment of the selected sliding window, therefore, we define the amplicon length formed by an odd number of fragments. Thus, we anchor the value ${\overline{S}}_{f}$ to the central fragment of sliding window, not to the abstract MSA locus.

From its previous value, the SMA can be obtained using the following recurrence formula:

\begin{eqnarray*}& & {\overline{S}}_{f}={\overline{S}}_{f-1}- \frac{{M}_{f-w}}{w} + \frac{{M}_{f}}{w} , \end{eqnarray*}

where ${\overline{S}}_{f}$ is the SMA value for a fragment ( *f*) of MSA; ${\overline{S}}_{f-1}$—the previous SMA value; *M*_*f*−*w*_—the number of mutations in the first fragment; *M*_*f*_—the number of mutations in the current sliding window after its shift by one fragment to the right.

### Web interface

MorphoCatcher uses a free “Agency” HTML5 page template from the “Start Bootstrap” service (https://startbootstrap.com) and supports the current releases of Chrome, Firefox, and Safari browsers. Computation time required for analysis usually takes few seconds depending on the server workload. The MorphoCatcher web service is freely available only for non-commercial and academic users at http://morphocatcher.ru. All output files can be downloaded as a ZIP-archive with the unique ID of the project. Graphic files have a publication quality and are available in different formats (*.eps, *.pdf, *.png, *.svg, and *.tiff).

### Command line version

A Python script of the MorphoCatcher tool can be used in the Linux terminal for large-scale research projects. A command line Python script of the tool is available at the GitHub public repository: https://github.com/shrshkv/MorphoCatcher.

### Manual and tutorial files

A detailed manual and tutorial files for two hypothetical case studies for the MorphoCatcher were developed to show the main applications and functions of the plugin tool during specific primer design. The case studies explain a simple bioinformatics protocol that can be used for the following example tasks: (i) to detect the bacterial potato pathogens from the *Dickeya* genus (Czajkowski et al., 2015) and (ii) distinguish two serotypes of potato virus Y (PVY) (Karasev & Gray, 2013) by the LAMP method. The manual demonstrates required file formatting and shows how to perform successful primer design using other well-known online bioinformatics services. Some recommendations were suggested for specific primer design using the alignment of orthologous target gene or whole-genome sequences. The manual and tutorial files are also available at https://github.com/shrshkv/MorphoCatcher.

### Primer design

After the alignment processing using MorphoCatcher, an output file from the “Input for PrimerExplorer” column that corresponds to the taxon of interest can be directly submitted to the PrimerExplorer 5.0 web server (Tomita et al., 2008; Eiken Chemical Co. LTD, Tokyo, Japan; http://primerexplorer.jp/e).

## Results

**Figure 1:**
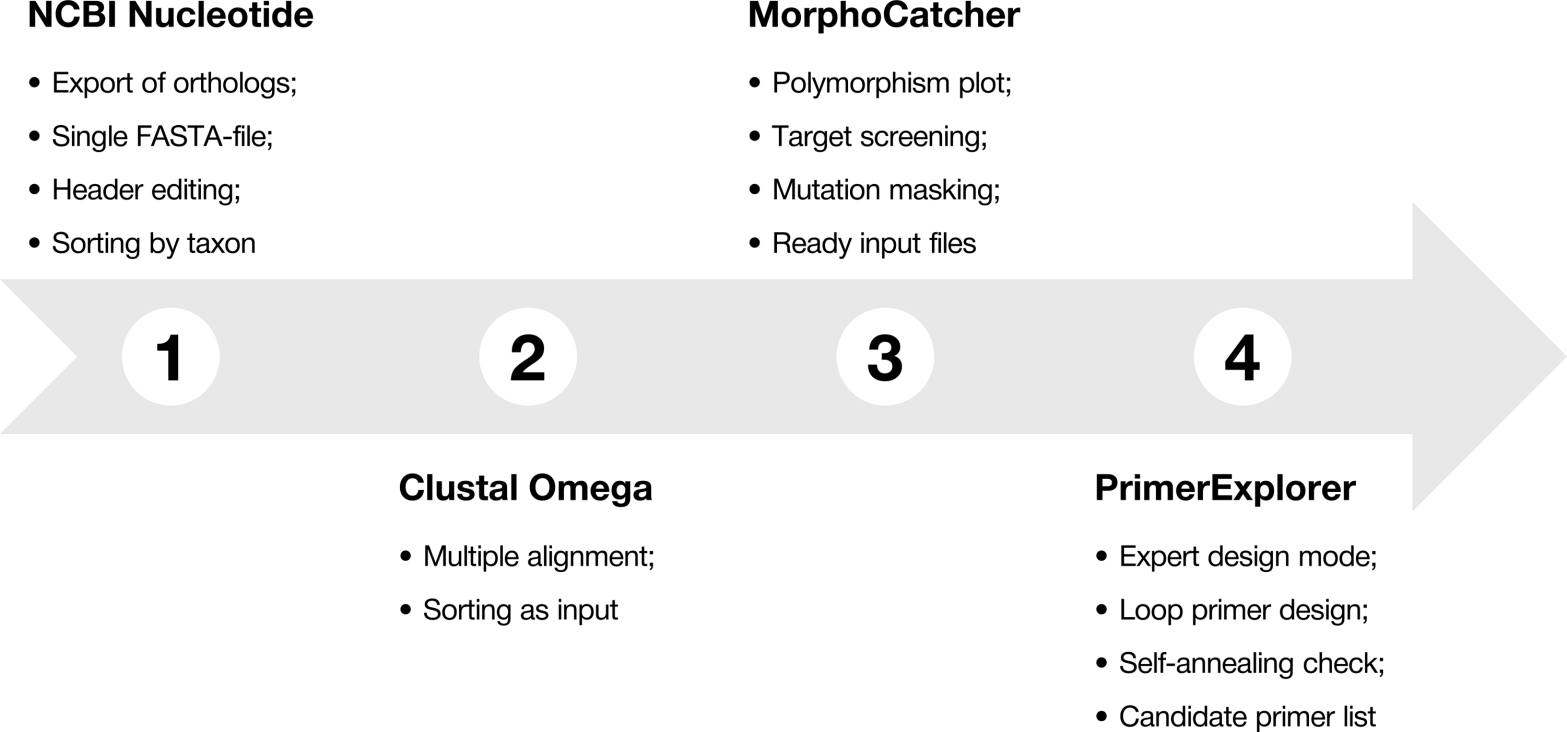
Protocol-at-a glance for successful design of taxon-specific primer sets.

To explain main functions of the MorphoCatcher, we describe a protocol for designing taxon-specific primers using the most widely-known bioinformatics online tools, including the NCBI Nucleotide database, NCBI BLAST, Clustal Omega, and PrimerExplorer (Fig. 1). The protocol contains several steps that help to find potential target sequence for diagnostic primers, keep the correct file formatting basing on well-known standards in bioinformatics, and obtain candidate primer sets for the LAMP experiments.

### The recommended MorphoCatcher protocol

**Step 1: defining closely related biological taxa.** First, the user should to collect several strains of the target species (or any other taxon) and the most closely related sequences from other species. The main idea of this step is to form groups of strains which should be distinguished by the LAMP diagnostics. These groups can be represented by different taxonomic ranks (e.g., species, genera, families, etc.), serotypes, or other groups of organisms. Total number of strains are usually limited by performance of the alignment tool used.

**Step 2: selecting target nucleotide sequences.** At this step, the user needs a set of potential targets in the taxon of interest, which can be obtained using the following services: ASAP (Glasner et al., 2006), Insignia (Phillippy et al., 2009), Panseq (Laing et al., 2010), and EDGAR (Blom et al., 2016). Optimal length of an amplicon for LAMP is about 130–200 bp, but sequences up to 300 bp are also acceptable (Notomi et al., 2000). Nevertheless, to obtain a wide variety of primer sets, we recommend the target size more than 1,000 bp. We also recommend to extract target sequence from the type strain with complete genome status.

**Step 3: exporting homologous sequences.** To provide statistically reliable taxon-specific mutations, different sequences should be extracted not only from the taxon of interest, but also from other closely related taxa. Related orthologous nucleotide sequences can be exported from the NCBI Nucleotide database (https://www.ncbi.nlm.nih.gov/nuccore), NCBI GenBank annotation of genome, or NCBI BLAST service (https://blast.ncbi.nlm.nih.gov) (Camacho et al., 2009).

To export orthologs using the NCBI BLAST service, a target nucleotide sequence should be used as the reference. The set of orthologous sequences can be saved from the NCBI BLAST output as a single FASTA text file. Then, the FASTA-header should be modified in accordance with two alternative formats:

>[Taxon ID]—[Target gene ID]—[Strain ID]

or

>[Taxon ID]—[Strain ID]

For example, the user can insert instead of [Taxon ID], [Target gene ID], or [Strain ID] any text that will be associated with organisms of interest.

To facilitate the rapid sequence searching on large datasets, a number of high-performance tools for multicore microprocessors can also be used, including PLAST (Nguyen & Lavenier, 2009), BLAST+ (Camacho et al., 2009), and DCBLAST (Yim & Cushman, 2017). The tools require some skills in command line of Linux computer systems.

Note, that if the taxon of interest belongs to insufficiently studied organisms and there are no orthologous sequences in the NCBI database, probably, the strategy of unique target search is more applicable for LAMP primer design.

**Step 4: making multiple sequence alignment.** To perform multi-species MSA of the orthologs, we recommend using the Clustal Omega service (https://www.ebi.ac.uk/Tools/msa/clustalo) (Sievers et al., 2011; Chojnacki et al., 2017). It is necessary to select an “Input” value of the “Order” menu to save the order of species in the input file. This point is important, because the MorphoCatcher algorithm recognizes several strains as belonging to the same species only if the strains have identical taxon ID in FASTA-header. The alignment should be saved as a text file.

As has been described in the previous step, the alignment step can also be accelerated by a local version of Clustal Omega algorithm and high-performance computing systems. The local run of Clustal Omega tool allows to overcome the web version limitations on input sequence number (up to 4,000 sequences or a maximum file size of 4 MB).

**Figure 2:**
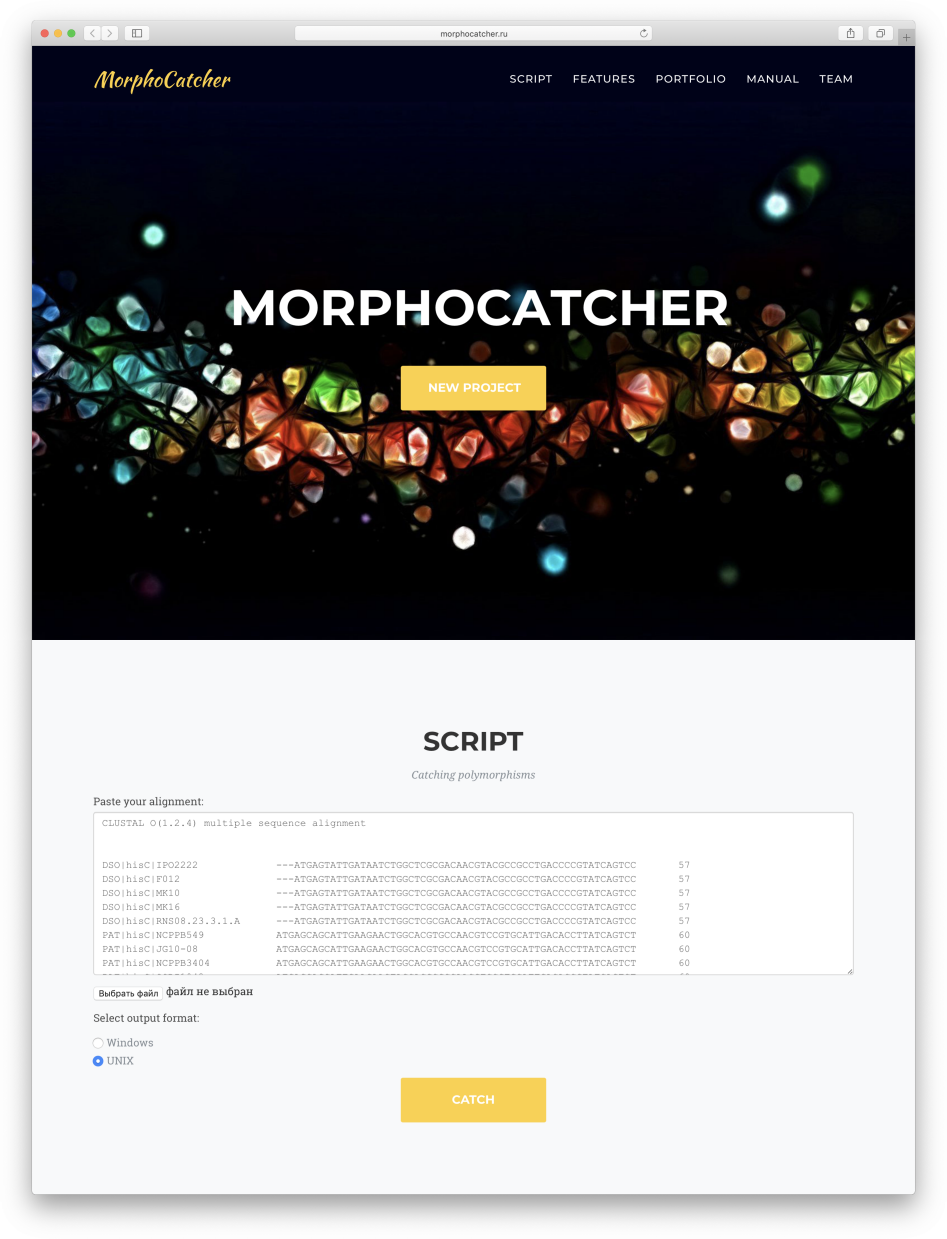
Web interface of the MorphoCatcher service with an alignment input bar and the output file format options.

**Step 5: masking taxon-specific mutations.** We suggest using the MorphoCatcher service (http://morphocatcher.ru) for processing of the MSA. The MorphoCatcher allows the user to paste or upload the alignment input file and select the output format, which depends on the operating system (e.g., Windows or UNIX). The MorphoCatcher web interface is shown in Fig. 2.

**Figure 3:**
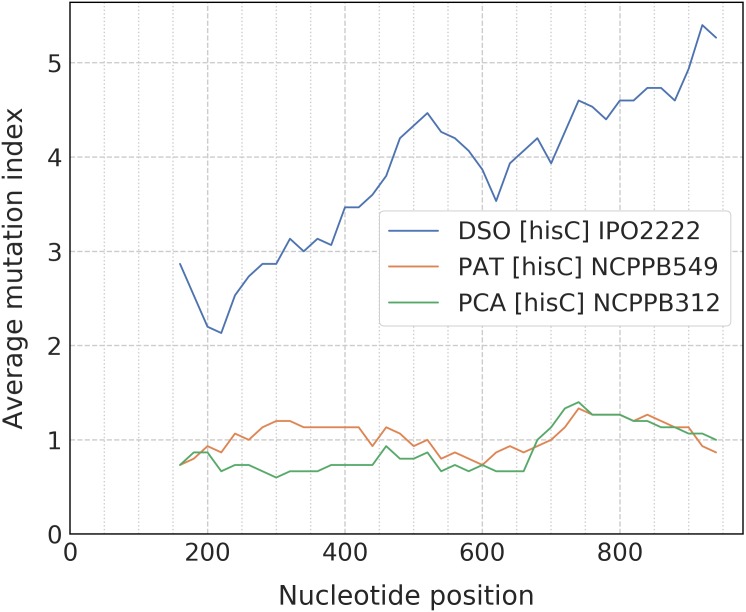
Sliding-window plot of the *hisC* gene polymorphism in closely related bacterial species. The legend abbreviations: DSO—the *Dickeya solani* species (IPO2222 is a type strain); PAT—the *Pectobacterium atrosepticum* species (NCPPB549 is a type strain); and PCA—the *P. carotovorum* species (NCPPB312 is a type strain). Peaks correspond to the high density of species-specific mutations. The plot was obtained after the alignment processing that contains up to five strains for each bacterial species.

When the alignment was uploaded, the user should click the “Catch” button which starts the processing of data to detect any taxon-specific mutations. For ease of polymorphism evaluation, we suggest the average mutation index that can be visualized as a sliding-window plot (Fig. 3). Regions with high density of taxon-specific mutations usually have a high value of the average mutation index. The peaks with maximal average mutation index and corresponding sequence can be selected as suitable locus for designing of specific primers. Then, if the target selected will be compatible with the design algorithm of PrimerExplorer, the most taxon-specific mutations will be included at the primer ends for better reaction specificity.

The most effective LAMP reactions can be performed with amplicons less than 300 bp (Notomi et al., 2000). Therefore, we use a sliding-window size of 300 bp in our algorithm. To obtain better primer specificity, we recommend to select nucleotide sequences with the average mutation index values from 2 and higher.

Once the MorphoCatcher execution of the alignment analysis is complete, the results tab appears with three columns that contain the following useful files:

• Processed MSA with highlighted species-specific mutations;
• Sliding-window plot of mutation density for each species of the alignment;
• Ready-to-use files for specific primer design using the PrimerExplorer service.

All abovementioned files can be downloaded from the MorphoCatcher output tab as a ZIP-archive. The user can also inspect positions of the species-specific mutations manually by using a processed alignment from the output files. The processed alignment file contains information about the number of species-specific mutations for each 20 nucleotides.

**Step 6: designing specific primers.** If the average mutation index is suitable for the taxon of interest, then the user can submit a corresponding text file from the “Input for PrimerExplorer” column directly to the PrimerExplorer 5.0 service (http://primerexplorer.jp/e). This file contains a target nucleotide sequence, conserved common region that is highlighted by asterisks (*), and detected taxon-specific mutations that highlighted by hyphen (-) symbols. These symbols mimic the PrimerExplorer format that was proposed to select any region of interest in the uploaded nucleotide sequence.

To design primer sets with a high density of taxon-specific mutations at 5′- or 3′-ends, the user should set the following parameters of the PrimerExplorer software:

• To select “Specific” mode of the “Primer option” menu;
• To click on the “Detail settings” button to turn on expert mode;
• To select a required GC-content of primers in the “Parameter condition” menu;
• To click on the “Generate” button;
• To set “Descending order” of primer sets in the “Sorting rule” menu;
• To click on the “Display” button.

**Figure 4:**
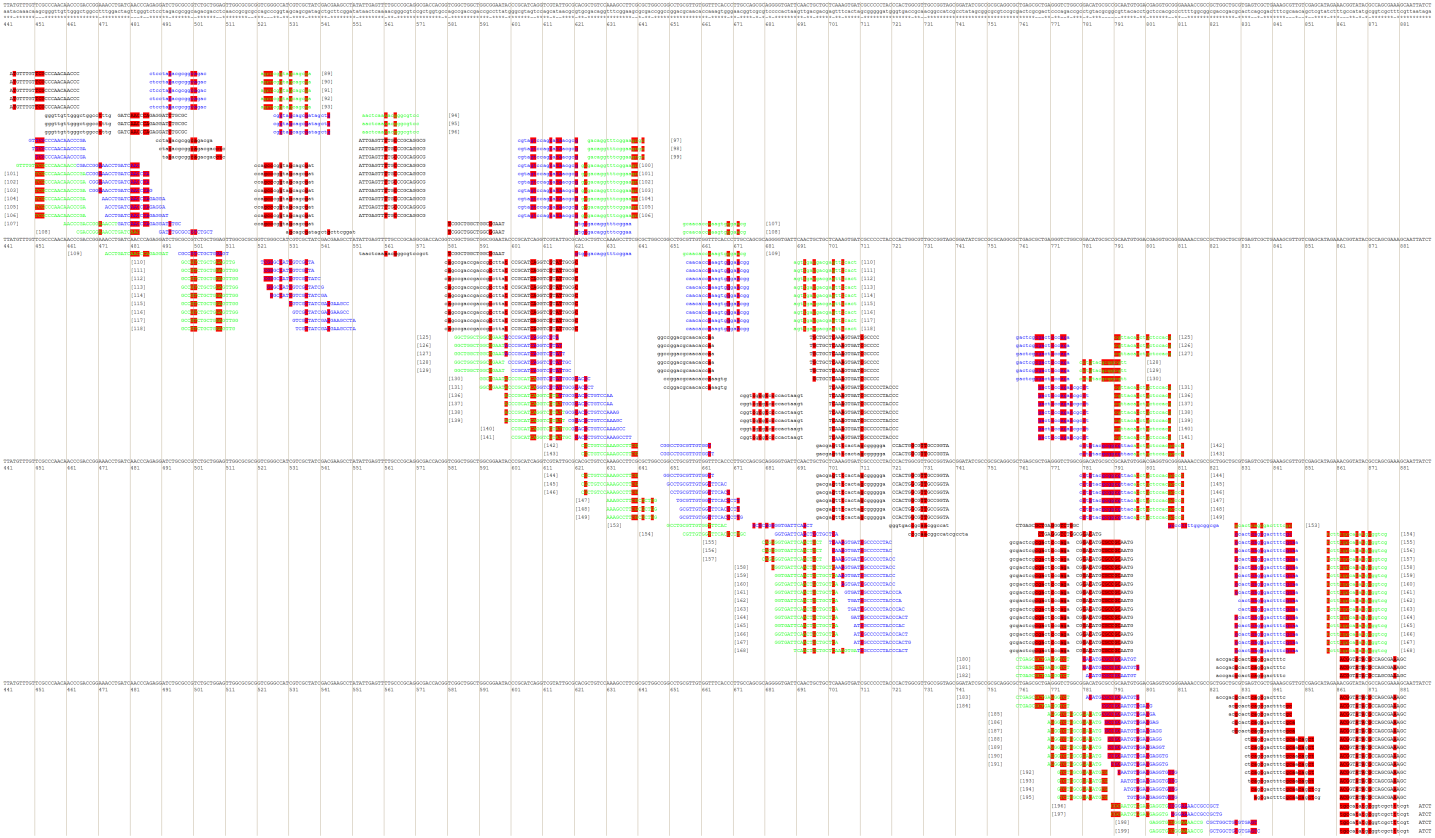
Part of the PrimerExplorer output page with different primer sets and their annealing sites. Detected species-specific mutations for the *Dickeya solani* strains are highlighted by red color. The mutations can be covered by the GC-rich oligos from different primer sets, therefore, various primer sets should be tested to verify specificity and sensitivity of the potential diagnostic assay.

After that, the user will be redirected to the page with various combinations of primer annealing sites, which can cover a different number of taxon-specific mutations (Fig. 4).

The MorphoCatcher protocol allows to solve a well-known problem of the PrimerExplorer caused by the limitation of the target sequence size up to 2,000 bp. This is possible due to our tool using an output file from the Clustal Omega algorithm that has no significant limit to the input sequence length. Thus, the user can evaluate the polymorphism level of target sequence preliminary and after that to select a suitable region (up to 2,000 bp) with high density of taxon-specific mutations for primer design.

So, after the last step of protocol the user can to select some primer sets for the oligo synthesis, reaction optimization, and assay validation basing on specificity and sensitivity tests. We recommend screening multiple primer sets in vitro, because at present there is no other effective way to predict which primer set will be recognized as a very successful primer design.

## Discussion

For LAMP method, a localization of taxon-specific SNPs at the primer ends can increase the reaction specificity and sensitivity. The most suitable regions of LAMP primers to place mutations are the following: 5′- and 3′-ends of inner (FIP and BIP) primers, as well as 3′-ends of outer (F3 and B3) and loop (LF and LB) primers. Thus, the PrimerExplorer service, as the most widely used software for LAMP primer design, has the potential to be enhanced by a simple plugin that improves the specific primer design mode using MSAs and allows to cover target sequence regions with high density of taxon-specific SNPs by the primer ends.

It is known that the most popular primer design strategy both for the PCR and LAMP experiments is based on the search of conserved common regions using MSAs. The strategy is also known as a primer design for group-specific amplification (Kalendar et al., 2017). We found that the strategy has one important disadvantage, because of some nucleotide sequences between the conserved common regions could be used to extend the search of potential annealing sites. In this work we suggest to find such nucleotides and use them as the target sites for coverage by the LAMP primer ends for increasing of the amplification specificity.

To simplify the search of taxon-specific polymorphisms using multiple alignment data, we develop the MorphoCatcher web service. The MorphoCatcher is a simple online plugin tool for the PrimerExplorer service that facilitates the processing of MSA composed from orthologous nucleotide sequences (e.g., bacterial or eukaryotic genes) or whole-genome sequences of viruses with conservative genome architecture (e.g., PVY strains and their recombinants).

In summary, the MorphoCatcher tool significantly extends the capability of the PrimerExplorer software. Comparison of well-established and useful bioinformatics tools for LAMP primer design is presented in Table 1.

There are some advantages and helpful applications of the MorphoCatcher software:

• The tool provides a unique capability for preliminary evaluation of taxonomic resolution, screening, and selection of target nucleotide sequence before the LAMP primer design step using a simple visualization approach based on sliding-window plots;
• The tool enables a monitoring of gene polymorphism or viral genome variability for different taxonomic ranks and any other group of organisms (e.g., serotypes). Such capability seems useful and promising as far as the data explosion from next-generation sequences in microbiology and epidemiology;
• All sequence and alignment files used in the MorphoCatcher protocol have widely used format (FASTA and ClustalW with nucleotide counts, respectively);
• The tool reduces time for searching of taxon-specific, or group-specific, mutations for LAMP experiments using PrimerExplorer.

### Future development

However, the MorphoCatcher also has some disadvantages. The protocol of primer design using the tool is not yet a real pipeline, therefore, we plan to improve the MorphoCatcher web service by modules for high-performance sequence similarity search and multiple alignment. We also plan to reduce the input from the user to a target sequence of interest in FASTA-format. It is will automatize further steps in according with the bioinformatics requirements to representativeness and reproducibility. We believe that these enhancements will lead to increasing of the user-friendliness and reduce complexity of the specific primer design in LAMP experiments.

**Table 1:** Comparison of bioinformatics tools for LAMP primer design.

Feature	Software			
	PrimerExplorer 5.0	MorphoCatcher	LAMP Designer 1.15	GLAPD
Platform	Web	Web, Linux	Windows, Macintosh	Linux
Command line	No	Yes	No	Yes
License	Free	Academic free	Commercial	Free
Target selection tool	No	Yes	No	No
Primer selection tool	Yes	No^a^	Yes	Yes
Loop primer design	Yes	Yes^a^	Yes	Yes
Specific design	Yes	Yes^a^	No	Yes
Common design	Yes	Yes^a^	No	Yes
Multiplex set design	No	No	Yes	No
Self-dimerization test	Yes	Yes^a^	Yes	Yes
BLAST test	No	No	Yes	No

**Notes.**

a These features were specified considering that MorphoCatcher uses the primer selection tool of PrimerExplorer.

## Conclusions

We have used the Python scripting language to develop the MorphoCatcher web tool. In summary, MorphoCatcher provides a simple protocol for molecular diagnostics community that accelerates the development of new taxon-specific LAMP primer sets. We believe that the MorphoCatcher web tool will be useful for those researchers who work with promising isothermal amplification methods, like LAMP and SHERLOCK (Gootenberg et al., 2017).

## Additional Information and Declarations

### Data Availability

The following information was supplied regarding data availability: Data is available at GitHub: https://github.com/shrshkv/MorphoCatcher.
